# Human and rat microsomal metabolites of *N*-*tert*-butoxycarbonylmethamphetamine and its urinary metabolites in rat

**DOI:** 10.1007/s11419-021-00595-6

**Published:** 2021-08-24

**Authors:** Hidenao Kakehashi, Takahiro Doi, Misato Wada, Tooru Kamata, Noriaki Shima, Akari Miyake, Atsushi Nitta, Ryutaro Asai, Shihoko Fujii, Shuntaro Matsuta, Keiko Sasaki, Hiroe Kamata, Hiroshi Nishioka, Akihiro Miki, Hiroshi Hasegawa, Munehiro Katagi

**Affiliations:** 1 Forensic Science Laboratory, Osaka Prefectural Police Headquarters, 1-3-18 Hommachi, Chuo-ku, Osaka, 541-0053 Japan; 2 Osaka Institute of Public Health Division of Hygienic Chemistry, Osaka, Japan; 3 Laboratory of Hygienic Sciences, Kobe Pharmaceutical University, 4-19-1 Motoyamakitamachi, Higasinada-ku, Kobe, Hyogo 658-8558 Japan

**Keywords:** *N*-*tert*-Butoxycarbonylmethamphetamine, Methamphetamine, Metabolite, Microsomes, Rat, LC–MS/MS

## Abstract

**Purpose:**

*N*-*tert*-Butoxycarbonylmethamphetamine (BocMA), a masked derivative of methamphetamine (MA), converts into MA under acidic condition and potentially acts as a precursor to MA following ingestion. To investigate the metabolism and excretion of BocMA, metabolism tests were conducted using human liver microsomes (HLM), rat liver microsomes (RLM) and rat.

**Methods:**

BocMA metabolites were analyzed after 1000-ng/mL BocMA incubation with microsomes for 3, 8, 13, 20, 30, and 60 min. Rats were administered intraperitoneal injections (20 mg/kg) of BocMA and their urine was collected in intervals for 72 h. Metabolites were detected by liquid chromatography–tandem mass spectrometry with five authentic standards.

**Results:**

Several metabolites including 4-hydroxy-BocMA, *N*-*tert*-butoxycarbonylephedrine and *N*-*tert*-butoxycarbonyl-cathinone were detected for HLM and RLM. In the administration test, three glucuronides of hydroxylated metabolites were detected. The total recovery values of BocMA and the metabolites during the first 72 h accounted for only 0.3% of the administered dose. Throughout the microsomal and administration experiments, MAs were not detected.

**Conclusion:**

Hydroxylation, carbonylation and *N*-demethylation were proposed as metabolic pathways. However, BocMA and phase I metabolites were hardly detected in urine. This study provides useful information to interpret the possibility of BocMA intake as the cause of MA detection in biological sample.

## Introduction

Methamphetamine (MA) is a substituted phenethylamine that is known as a highly addictive stimulant. It has been abused worldwide and its smuggling operations are sophisticated [1, 2]. In recent years, viscous liquids containing *N*-*tert*-butoxycarbonylmethamphetamine (Boc-protected MA: BocMA) disguised as essential oils and hair products have been seized at several international airports [3]. The Boc group has been commonly used for protecting primary and secondary amines in organic synthesis. Boc protection is generally stable against heat, bases, and oxidation, whereas they can be easily de-protected by treatment with strong acids to produce the desired compound in excellent yield [4–7]. Kurakami et al. reported that BocMA converted quickly into MA in heated hydrogen chloride aqueous solution (HCl*aq*) at good yield [6]. BocMA is of increasing concern, acting as new smuggling operation tools due to the masked chemical structure that circumvents legal restrictions. In addition, previous studies showed that BocMA yields MA by exposure to simulated gastric juice including diluted HCl*aq* at 37 °C (body temperature), making it an excellent potential precursor drug following oral administration [7]. Collins et al. reported that *N*-*tert*-butoxycarbonylmethylenedioxymethamphetamine (BocMDMA) seized in Australia showed the same potential of being a precursor drug for MDMA [8]. In other words, Boc-masked phenethylamines can be abused as a tricky but clever way to circumvent the legal restrictions. For avoiding wrongful arrests, it becomes crucial to discriminate Boc-protected drug administration that of original drug MA.

Metabolism of termed “precursor drug” to MA has been described for several compounds including benzphetamine, famprofazone and selegiline [2, 9–13]. Previous studies have investigated their metabolism and excretion, clarifying that specific metabolites, proportion of MA to amphetamine, and enantiomers of MA are useful indicators for interpreting the origin of MA in urine samples. Thus, proving BocMA administration also requires a scientific evidence based on a study of BocMA absorption, metabolism, and excretion.

In our previous study, we evaluated the stability of BocMA in model gastric juice solution (pH 1.5, 37 °C), and showed its gradual decomposition with a half-life of 50 min [7]. This result indicated that some portion of the orally ingested BocMA can pass through the stomach into the intestine without converting into MA, and undergo absorption followed by first-pass effect in the liver. However, to the best of our knowledge, no such study of BocMA metabolism and excretion has been reported. In this study, we explored the in vitro and in vivo metabolites of BocMA obtained from samples of human liver microsomes (HLM), rat liver microsomes (RLM) incubation, and rat administration using liquid chromatography–quadrupole time-of-flight mass spectrometry (LC–QToF–MS) for qualitative analysis and liquid chromatography–triple quadrupole mass spectrometry (LC–tripleQ–MS) for quantitative analysis. From the obtained results, we will discuss the metabolism and excretion to propose urinary metabolites for proving BocMA ingestion in human.

## Materials and methods

### Reagent and rat

Fifty donor HLM pool, RLM, NADPH system solution A and NADPH system solution B were obtained from Corning (Corning, NY, USA), and tris(hydroxymethyl)aminomethane buffer pH 7.4 was from NIPPON GENE (Tokyo, Japan). Four 6-week-old male Kwl: Wistar rats (200 ± 20 g) were from Kiwa Laboratory Animals (Wakayama, Japan). Prior to testing, rats were kept at 23–25 °C on a 12-h light–dark cycle with access to food and water ad libitum. β-Glucuronidase (*Escherichia coli*, Type IX-A, activity: 4,040,000 units/g) was purchased from Sigma-Aldrich (St. Louis, MO, USA). Positive Calibration solution for QToF–MS calibration procedure was from AB Sciex (Concord, ON, Canada) and all other chemicals and reagents of analytical grade or quality were from FUJIFILM Wako Pure Chemical (Osaka, Japan).

### Synthesis of authentic standards

The authentic standards including BocMA, six metabolite candidates M1–D1, M1–D2, M2, M3, M4, Boc-protected methcathinone (BocMC) and internal standard (IS, *N-*valerylmethamphetamine) were chemically synthesized as shown in procedures (1)–(7). All standards were purified with silica gel column chromatography followed by identification using nuclear magnetic resonance spectroscopy (NMR). Cathinone and methcathinone as ingredients were synthesized by reference to the previous reports [14–16]. All standard stock solutions were prepared in acetonitrile and adjusted to working concentrations with distilled water immediately prior to use.

1. BocMA. To a solution of *d-*MA hydrochloride (450 mg) in tetrahydrofuran (THF, 10 mL), triethylamine (2 mL) and 4-dimethylaminopyridine (DMAP, cat.) were added. After stirring at room temperature for 10 min, di-*t*-butyl dicarbonate (Boc_2_O) (880 mg) was added and the resulting mixture was additionally stirred overnight. The mixture was diluted with water and extracted with ethyl acetate. The combined organic layer was washed with saturated aqueous solution dried over Na_2_SO_4_, and evaporation of the solvent left a slight yellow oil. Purification by column chromatography gave the Boc-protected product in 94% yield. ^1^H NMR (400 MHz, CD_3_OD, 50 °C; BocMA): δ 7.24 (t, *J* = 7.2 Hz, 2H), 7.16 (m, 3H), 4.40 (br, 1H), 2.74 (m, 2H), 2.70 (s, 3H), 1.32 (s, 9H), 1.18 (d, *J* = 7.2 Hz, 3H).
2. *N-tert-*butoxycarbonylephedrine (M1–D1), *N-tert-*butoxycarbonylpseudoephedrine (M1–D2). By reference to (1) in this section, synthesis from ephedrine hydrochloride and pseudoephedrine hydrochloride afforded M1–D1 (50% yield) and M1–D2 (51% yield), respectively. ^1^H NMR (400 MHz, CDCl_3_, 50 °C; M1–D1): δ 7.36–7.26 (m, 4H), 7.24–7.22 (br, 1H), 4.78 (s, 1H), 4.11 (t, *J* = 6.2 Hz, 1H), 2.65 (br, 3H), 1.39 (s, 9H), 1.22 (d, *J* = 6.8 Hz, 3H). (400 MHz, CDCl_3_, 50 °C; M1–D2): δ 7.36–7.25 (m, 5H), 4.54 (d, *J* = 6.8 Hz, 1H), 4.15 (m, 1H), 2.74 (br, 3H), 1.45 (s, 9H), 1.02 (d, *J* = 7.2 Hz, 3H).
3. *N-tert-*butoxycarbonylamphetamine (M2). By reference to (1) in this section, amphetamine hydrochloride afforded M2 (99% yield). ^11^H NMR (400 MHz, CDCl_3_, 50 °C; M2): δ 7.29–7.14 (m, 5H), 3.89 (br, 1H), 2.82–2.65 (m, 4H), 1.42 (s, 9H), 1.07 (d, *J* = 6.8 Hz, 3H).
4. *N-tert-*butoxycarbonyl-4-hydroxymethamphetamine (M3). To a solution of 4-hydroxymethamphetamine sulfate (3 g) in THF, triethylamine (3.9 mL) was added. After stirring at room temperature for 20 min, *tert-*butyldimethylsilyl chloride (TBDMSCl, 2.3 g) was added and the resulting mixture was additionally stirred overnight. The mixture was diluted with water and extracted with ethyl acetate. The combined organic layer was washed with saturated aqueous solution dried over Na_2_SO_4_, and evaporation of the solvent left a colorless oil. Purification by column chromatography gave a synthetic intermediate (4TBDMSO-MA) in 50% yield. By reference to (1) in this section, synthesis from the intermediate afforded 4TBDMSO-BocMA (66% yield). To a solution of 4TBDMSO-BocMA (500 mg) in THF (10 mL), tetrabutylammonium fluoride (500 mg) was added, and the resulting mixture was stirred at room temperature for 10 min. The mixture was diluted with water and extracted with ethyl acetate. The combined organic layer was washed with saturated brine and dried over Na_2_SO_4_, and evaporation of the solvent left a colorless oil. Purification by column chromatography gave M3 as white solid in 77% yield. ^1^H NMR (400 MHz, CDCl_3_, 50 °C; M3): δ 7.10 (d, *J* = 8.0 Hz, 2H), 6.72 (d, *J* = 8.0 Hz, 2H), 5.28 (t, *J* = 6.4 Hz, 1H), 2.71–2.55 (m, 5H), 1.36 (s, 9H), 1.12 (d, *J* = 6.4 Hz, 3H).
5. *N*-*tert*-butoxycarbonyl-2-amino-1-phenylpropan-1-one (Boc-protected cathinone, M4). By reference to (1) in this section, synthesis from cathinone afforded M4 (89% yield). ^1^H NMR (400 MHz, CDCl_3_, 50 °C; M4: δ 7.96 (d, *J* = 7.2 Hz, 2H), 7.58 (t, *J* = 7.2 Hz, 1H), 7.48 (t, *J* = 7.2 Hz, 2H), 5.27 (br, 1H), 1.45(s, 9H), 1.39 (d, *J* = 7.6 Hz, 3H).
6. *N-tert-*butoxycarbonyl-2-(methylamino)-1-phenylpropan-1-one (BocMC). By reference to (1) in this section, synthesis from methcathinone afforded BocMA (80% yield). ^1^H NMR (400 MHz, CDCl_3_, 50 °C; M4): δ 7.88 (br, 2H), 7.45 (t, *J* = 7.2 Hz, 2H), 7.35 (t, *J* = 7.2 Hz, 2H), 5.59 (br, 1H), 2.70–2.57 (m, 3H), 1.36 (s, 9H), 1.19 (s, 3H).
7. *N-*valerylmethamphetamine (IS). To a solution of MA hydrochloride (100 mg) in THF (10 mL), valeric anhydride (200 mg) followed by triethylamine (0.45 mL) and DMAP was added, and the resulting mixture was refluxed for 1 h. The mixture was diluted with water and extracted with ethyl acetate. The combined organic layer was washed with saturated aqueous solution dried over Na_2_SO_4_, and evaporation of the solvent left a yellowish residue. Purification by column chromatography gave *N-*valerylmethamphetamine (IS) in 41% yield. ^1^H NMR (400 MHz, CDCl_3_; *N-*valerylmethamphetamine): δ 7.21–7.09 (m, 5H), 4.08 (m, 1H), 2.85–2.71 (m, 5H), 2.17 (t, *J* = 7.2 Hz, 1H), 2.05–1.85 (m, 1H), 1.45–1.08 (m, 7H), 0.83 (m, 3H).

### Microsomal metabolism test

BocMA was incubated at 1 μg/mL with human (50 donor-pooled) or rat (pooled) liver microsomes at 37 °C in triplicate. In a 1.5-mL tube, 2 μL of BocMA solution in acetonitrile (500 μg/mL), 888 μL of Tris-buffer (0.1 M, pH 7.4), 50 μL of NADPH regenerating system (NRS) solution A and 10 μL of NRS solution B were mixed. After the addition of HLM (protein: 20 mg/mL, 50 μL) or RLM (protein: 20 mg/mL, 50 μL), the mixture was incubated with gentle shaking. A 100-μL aliquot was removed at 0, 3, 8, 13, 20, 30 and 60 min and extracted with 500 μL of pre-iced chloroform including IS (20 ng/mL). The organic layer was dried under nitrogen atmosphere. The residue was dissolved in 100 μL of 20% methanol. After centrifuging at 7000×*g* for 10 min, a 10-μL aliquot of the supernatant was analyzed by LC–MS/MS.

The half-life and subsequent microsomal intrinsic clearance (Cl_int,mic_) were calculated from plots of BocMA concentrations against time. Cl_int,mic_ was scaled to whole liver size to obtain hepatic intrinsic clearance (CL_int_) for human and rat. Hepatic clearance (CL_H_) and extraction ratio (ER) were estimated based on the well-stirred model without considering protein binding [17]. Microsomal protein per gram of liver (human: 45.0 mg/g, rat: 44.8 mg/g), liver weight per kilogram of body weight (human: 20 g/kg, rat: 40 g/kg) and liver blood flow (human: 20.7 mL/min/kg, rat: 55.2 mL/min/kg) were used for the calculations [17–19].

### Rat administration test

After a week quarantine in metabolic cages, rats were administered intraperitoneal injections (i.p.) of 20 mg/kg BocMA dissolved in vehicle solution comprising 1% polyoxyethylene sorbitan monolaurate (Tween 20). At 4, 8, 24, 48, and 72 h post injection, excreted urine in a receptacle (100 μL of toluene added in advance) was collected and stored at − 20 °C until analysis. Rats were euthanized by peritoneal injection of 200 mg/kg pentobarbital sodium (i.p.) at 72 h post injection.

To a 200-μL rat urine sample was added 20 μL of IS solution (200 ng/mL) and the mixture was extracted three times with 200 μL chloroform. The combined organic layer was dried under nitrogen atmosphere. The residue was dissolved in 100 μL of 20% methanol. After centrifuging at 7000×*g* for 10 min, a 10-μL aliquot of the supernatant was injected into an LC–MS/MS system for the phase I metabolites determination. The rat urine sample was added of β-glucuronidase (20,200 units/mL urine) without pH adjustment, and was incubated at 37 °C for 90 min to hydrolyze the conjugates prior to extraction as necessary.

To a 100 μL of rat urine sample, 300 μL of ice-cold methanol was added. After mixing by vortex, the mixture was centrifuged (9000×*g*, 5 °C, 10 min). The supernatant was double-diluted with distilled water. A 10-μL aliquot was injected into an LC–MS/MS system for the phase I and II metabolites qualitative analysis. The phase II metabolites were analyzed based on expected metabolites, such as glucuronide and sulfate conjugates, of hydroxylated phase I metabolites.

### Analytical instruments and condition

1. NMR

Synthesized compounds were identified with ECZ-400S obtained from JEOL (Tokyo, Japan). Analytical condition was as follows: irr domain, ^1^H; irr frequency, 400 MHz; solvent, CDCl_3_ or CD_3_OD; temperature, 25 or 50 °C; range, − 2.5 to 12.5 ppm.

(2) LC–MS/MS
(3) LC analysis was conducted on a Prominence Series UFLC system (Shimadzu) using L-column2 ODS semi-micro-column (150 mm × 1.5 mm i.d., 5 μm particles; Chemicals Evaluation and Research Institute, Tokyo, Japan). The analytes were chromatographed by linear gradient elution with (A) 10 mM ammonium acetate buffer (pH 5); and (B) methanol at a flow rate of 0.1 mL/min and 40 °C column temperature. A gradient was applied starting from 95% A/5% B, and linearly increased to 5% A/95% B over 15 min, and held for 10 min.
(4) LC–QToF–MS analysis was conducted on a Triple TOF 5600 hybrid quadrupole time-of-flight tandem mass spectrometer (AB Sciex, Concord, ON, Canada) equipped with an electrospray ionization (ESI) interface. The MS conditions were as follows: ion spray voltage, 5.5 kV; turbo spray temperature, 200 °C; de-clustering potential (DP), 40 V; collision energy (CE), 10 eV. Nitrogen was used as the nebulizer and collision gas. Full scan was run in the positive mode with a mass range from *m*/*z* 100–1000 and with a 250-ms accumulation time. For the information-dependent acquisition criteria, the 20 most intense ions that exceeded 100 count per sec were selected to perform a product ion scan, and the ion scan ranged from *m*/*z* 50–1000 with a 50 ms accumulation time. The instrument was mass-calibrated prior to analysis, infusing a Positive Calibration Solution (AB Sciex) at a flow rate of 100 μL/min on an automated calibration delivery system. Quantitative analysis (LC–tripleQ–MS) was performed on a Prominence Series UFLC system linked to an API 3200 QTRAP hybrid triple quadrupole linear ion-trap mass spectrometer (AB Sciex) equipped with an ESI interface in the selected reaction monitoring (SRM) mode. Nitrogen was used as the nebulizer and collision gas, and the SRM parameters for each compound are shown in supplementary material.

### Sample preparation for method validation

1. Microsomes

To 99 μL of the microsomal mixture prepared as previously shown without BocMA, 1 μL of 0.01–100 μg/mL analytes (BocMA, M1–D1 and M1–2) solution was added. The mixture was extracted three times with 500 μL of pre-iced chloroform with IS (20 ng/mL). The organic layer was dried under nitrogen atmosphere. The residue was dissolved in 100 μL of 20% methanol. After centrifuging at 7000×*g* for 10 min, a 10-μL aliquot of the supernatant was analyzed by LC–MS/MS for calibration curves. Quality control samples were prepared by spiking authentic standards at concentration of 40, 400 ng/mL (*n* = 5).

(2) Urine

To 990 μL urine from rat injected with vehicle, 10 μL of 0.01–10 μg/mL analytes and IS solution was added. The mixture was extracted three times with 500 μL of pre-iced chloroform. The organic layer was dried under nitrogen atmosphere. The residue was dissolved in 100 μL of 20% methanol. After centrifuging at 7000×g for 10 min, a 10-μL aliquot of the supernatant was analyzed by LC–MS/MS for calibration curves. Quality control samples were prepared by spiking authentic standards at concentration of 4, 40 ng/mL (*n* = 5).

### Method validation

Quantitation of BocMA M1–D1and M1–D2 in microsomes mixture and urine was validated by the LC–MS/MS procedure described in the experimental section. LC–MS/MS analyses exhibited high degree of linearity (microsomes: *r*^2^ > 0.993, urine: *r*^2^ > 0.995) throughout the calibrator concentration range from 10 to 1000 ng/mL for microsomes, and 0.3 to 100 ng/mL for urine.

When the urinary analyte concentration values were above the calibration range, concentrations were determined after diluting sample with a drug-free urine to the appropriate concentration within the calibration range. Both the limit of detection (LOD) and limit of quantification (LOQ) were defined as the detection limits of the target ion peaks on each extracted ion chromatogram in the SRM mode (SN ≥ 3).

## Results

### MS condition

BocMA experienced protonation and significant decomposition through ESI and in-source collision-induced dissociation (CID) of LC–MS/MS under a widely used analytical condition for low-molecular compound (turbo spray temperature: 500 °C, DP: 60 V). This condition remarkably de-protected BocMA and produced protonated MA [C_10_H_16_N]^+^ at *m*/*z* 150.1 (data not shown). This phenomenon was also observed in the analysis of BocMA metabolites. Analytical parameters were optimized by varying the turbo spray temperature (100–500°C) and DP (20–80 V). The ion intensity of protonated BocMA [C_15_H_24_NO_2_]^+^ at *m*/*z* 250.2 was monitored with varied parameters; the intensity was highest under the low-energy condition (turbo spray temperature: 200 °C and DP: 40) due to suppressed decomposition. All Boc-protected compounds were thus analyzed under this optimized condition. Quantitative analytical parameters for the 3200 QTRAP system were also optimized to detect the analytes at the highest sensitivity by selecting the protonated molecules as precursor ions. Boc-protected compounds can lead to mis-identification due to de-protection of the Boc group under ESI and in-source CID, requiring analytically optimized parameters on each mass spectrometer to detect the protonated analyte.

### Method validation

LODs and LOQs for both BocMA and M1 (D1, 2) in the microsomal reaction mixture were 1 and 10 ng/mL, respectively. For rat urine sample, LODs were 0.1 ng/mL and LOQs were 0.3 ng/mL for all compounds. Accuracy and precision values were < 10% for all compounds at all examined concentrations. The concentrations of BocMA and M1 (D1, 2) in microsomes and urine samples were quantitated using the above validated LC–MS/MS procedure and calibration curves.

### Microsomal metabolism

1. Degradation and kinetic parameters
  To evaluate the hepatic metabolism of BocMA after absorption in human, we performed metabolism tests using human and rat liver microsomes for obtaining half-lives and kinetic parameters. BocMA concentration depleted in both human and rat liver microsomal incubations, and their half-lives were calculated to be 3.2 min for HLM and 2.0 min for RLM. According to the half-lives, the kinetic parameters were obtained as shown in Table 1. CL_int_ (200 mL/min/kg) of HLM suggested that BocMA is notably susceptible to hepatic metabolism in comparison with 29 drugs (CL_int_: < 0.52 and 0.9–189 mL/min/kg, average: 34 mL/min/kg) tested by Obach. [17]. ER values, 0.90 for HLM and 0.92 for RLM also suggested that BocMA experiences a significant first-pass effect after absorption.
  (2) Microsomal metabolites

**Table 1:** Half-lives and pharmacokinetic parameters of BocMA incubated in human liver microsomes (HLM) and rat liver microsomes (RLM)

Pharmacokinetic parameter	HLM (mean ± SD, *n* = 3)	RLM (mean ± SD, *n* = 3)
Half-life (min)	3.2 ± 0.3	2.0 ± 0.2
CL_int,micr_ (mL/min/mg)	0.22 ± 0.02	0.36 ± 0.04
CL_int_ (mL/min/kg body wt.)	200 ± 20	640 ± 70
CL_H_ (mL/min/kg body wt.)	19 ± 1	51 ± 1
ER	0.90 ± 0.01	0.92 ± 0.01

*CL*_*int,micr*_ intrinsic microsome clearance, *CL*_*int*_ estimated intrinsic clearance, *CL*_*H*_ estimated hepatic clearance, *ER* extraction ratio

**Fig. 1:**
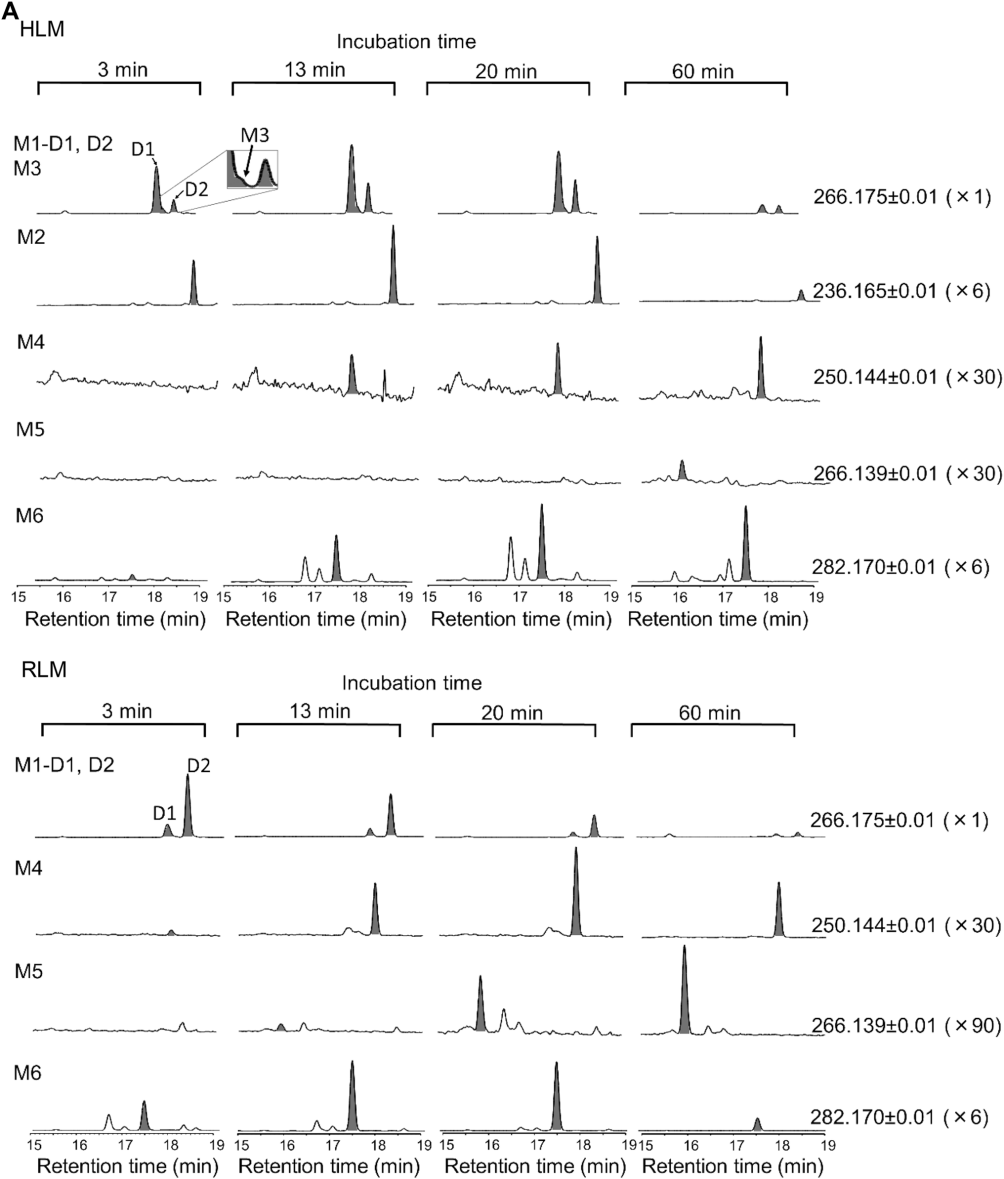

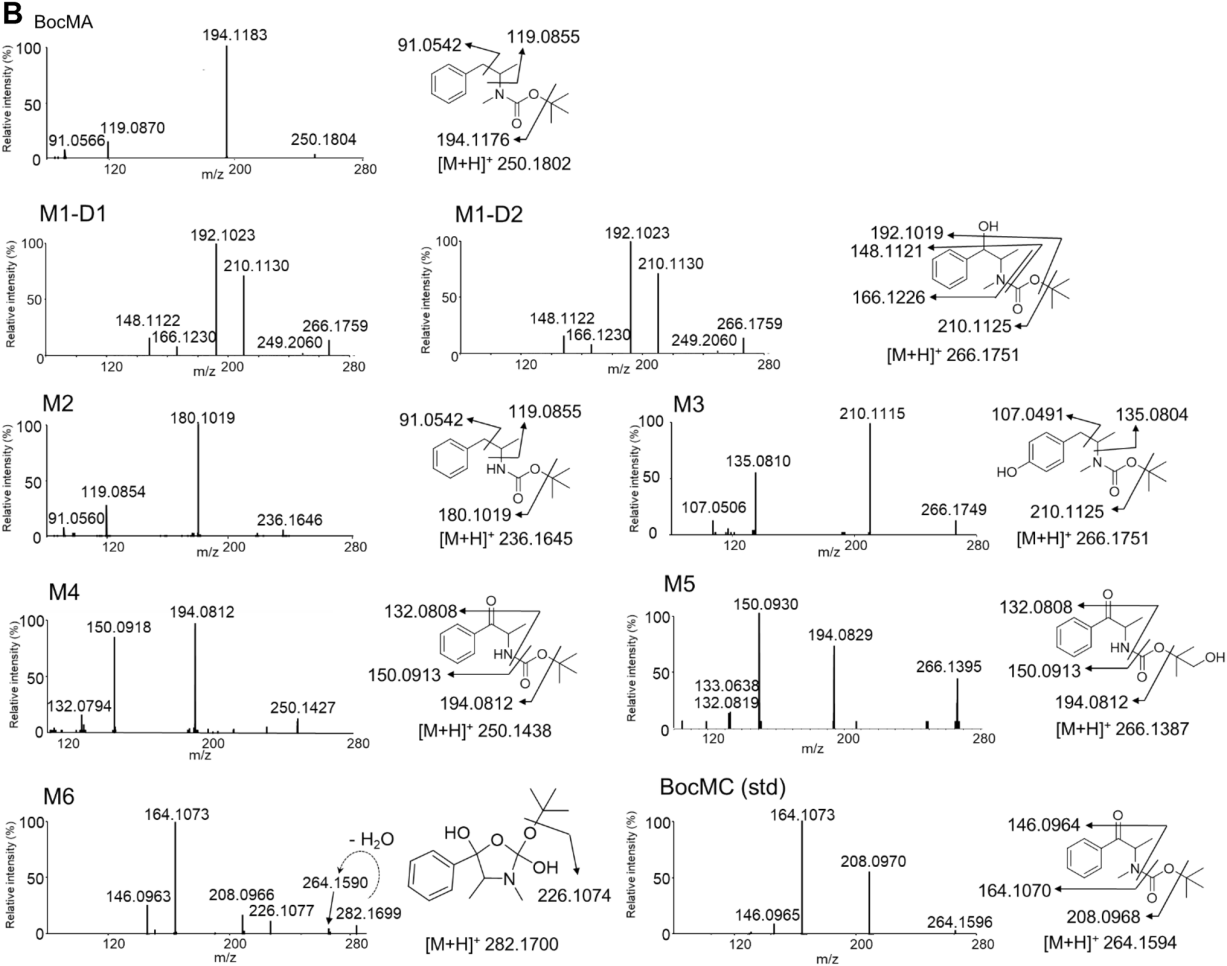
**A** The extracted ion chromatograms of BocMA metabolites following incubation with human liver microsomes (HLM) and rat liver microsomes (RLM). **B** Product ion spectra of the microsomal metabolites (M1–6) and authentic standards (BocMA, BocMC)

To investigate the metabolic pathways of BocMA, we explored its metabolites by LC–QToF–MS after HLM and RLM incubation. In both HLM and RLM tests, MA and its metabolites, such as amphetamine (AP), 4-hydroxy methamphetamine (4OHMA) and 4-hydroxy amphetamine (4OHAP), were not detected. BocMA (retention time: 20.1 min) after HLM incubation produced seven metabolite candidates, such as M1–D1, M1–D2, M2, M3, M4, M5 and M6, eluting at 18.0, 18.5,19.0, 18.3, 17.9, 15.9 and 17.5 min, respectively (Fig. 1A). Their product ion spectra are displayed in Fig. 1B. For RLM, five candidates M1–D1, M1–D2, M4, M5 and M6 were detected (Fig. 1A, B). Their chemical structures were extrapolated, respectively, from accurate masses of the protonated molecules, product ion spectra, and retention times. We were able to synthesize five of the candidates (M1–D1, M1–D2, M2, M3 and M4) as authentic standards as shown in the methods section. These authentic standards allowed identifications of M1–D1 (Boc-ephedrine), M1–D2 (Boc-pseudoephedrine), M2 (Boc-AP), M3 (Boc-4OHMA) and M4 (Boc-cathinone). Despite the lack of authentic standard, M5 was proposed as *tert-*butyl hydroxylated M4 because M5 displayed a protonated molecule [M + H]^+^ at *m*/*z* 266.1395 indicating the molecular formula as C_14_H_19_NO_4_, and product ions at *m*/*z* 194.0829, 150.0930, and 132.0819 as shown in Fig. 1B with assignments. Additionally, M6 was also characterized as an intermediate metabolite between M1 and M4. Chemical structures of M1 and M4 implied that M1 was bio-transformed into M4 via an intermediate including Boc-protected nor-ephedrine and/or Boc-protected methcathinone (BocMC), but these two desired compounds were not detected in any of the microsomal tests. Alternatively, M6 was proposed as the intermediate because of its protonated molecule [M + H]^+^ at *m*/*z* 282.1699 indicating the molecular formula as [C_15_H_23_NO_4_], which can be rearranged to [C_15_H_21_NO_3_ + H_2_O] of the monohydrated BocMC. Moreover, protonated M6 underwent dehydration (minus H_2_O: minus 18.0109) to produce a product ion at *m/z* 264.1590 as [C_15_H_21_NO_3_] close to the protonated BocMC [M + H]^+^ at *m*/*z* 264.1594 with mass error (1.5 ppm). In addition, M6 produced product ions at *m*/*z* 208.097, 164.107, and 146.096 that coincide with those of BocMC, showing a substantial correlation between M6 and BocMC.

**Fig. 2:**
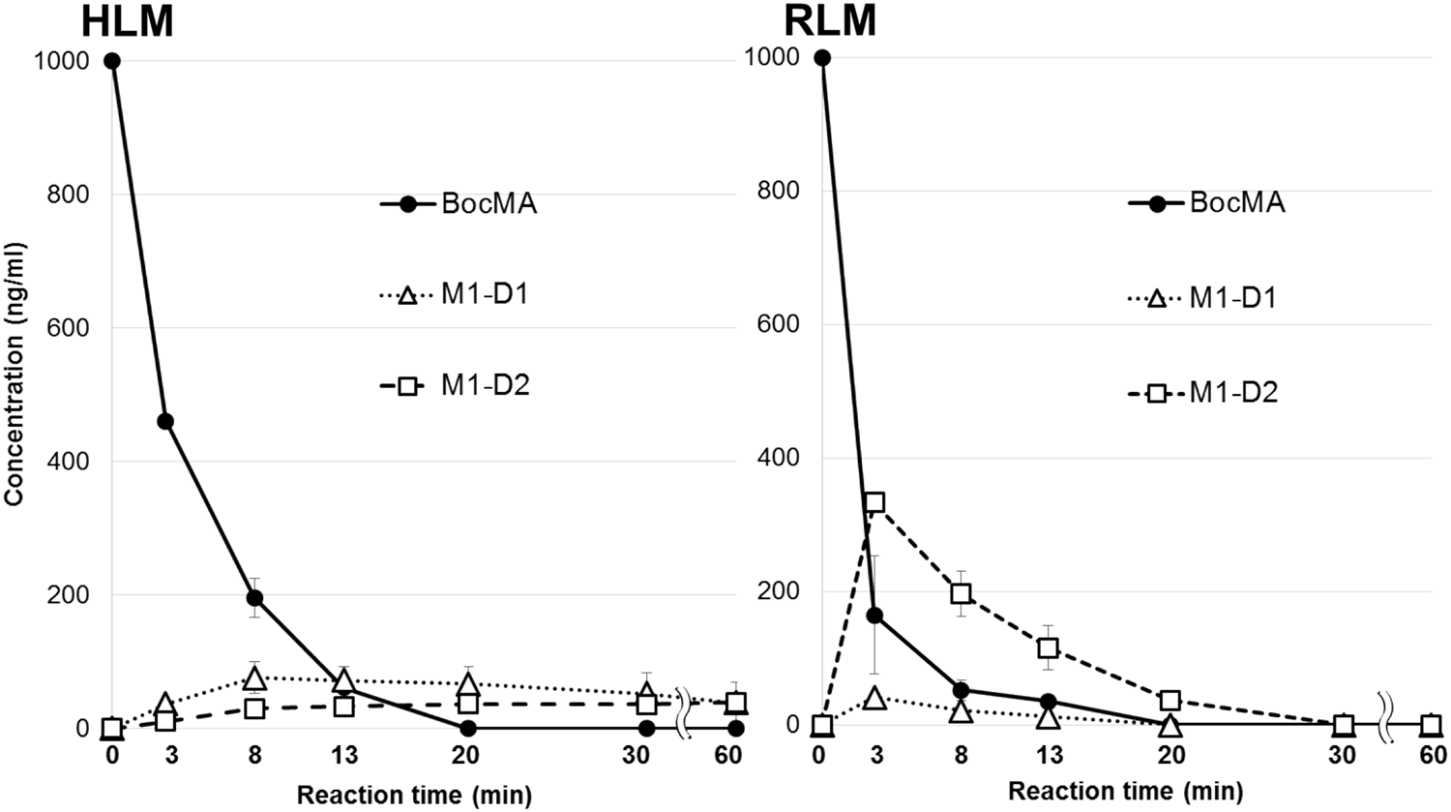
Concentrations of BocMA and M1 (D1, 2) following incubation of 1000-ng/mL BocMA with human liver microsomes (HLM) and rat liver microsomes (RLM), *n* = 3

M1 showed the highest peak intensity among the microsomal metabolites of BocMA, suggesting its contribution to the major metabolic pathway. To evaluate the contribution, we quantified M1 in each incubation time (Fig. 2). In RLM test, 83% of BocMA was degraded in the first 3 min from the reaction beginning, and 45% of the degraded products were detected as M1–D1 (5%) and M1–D2 (40%). In HLM test, 80% of BocMA was also degraded in the first 8 min, and 14% of the degraded products were detected as M1–D1 (10%) and M1–D2 (4%). These results indicate the major metabolic pathway of BocMA to be hydroxylation at the benzyl position.

### Urinary metabolites

**Fig. 3:**
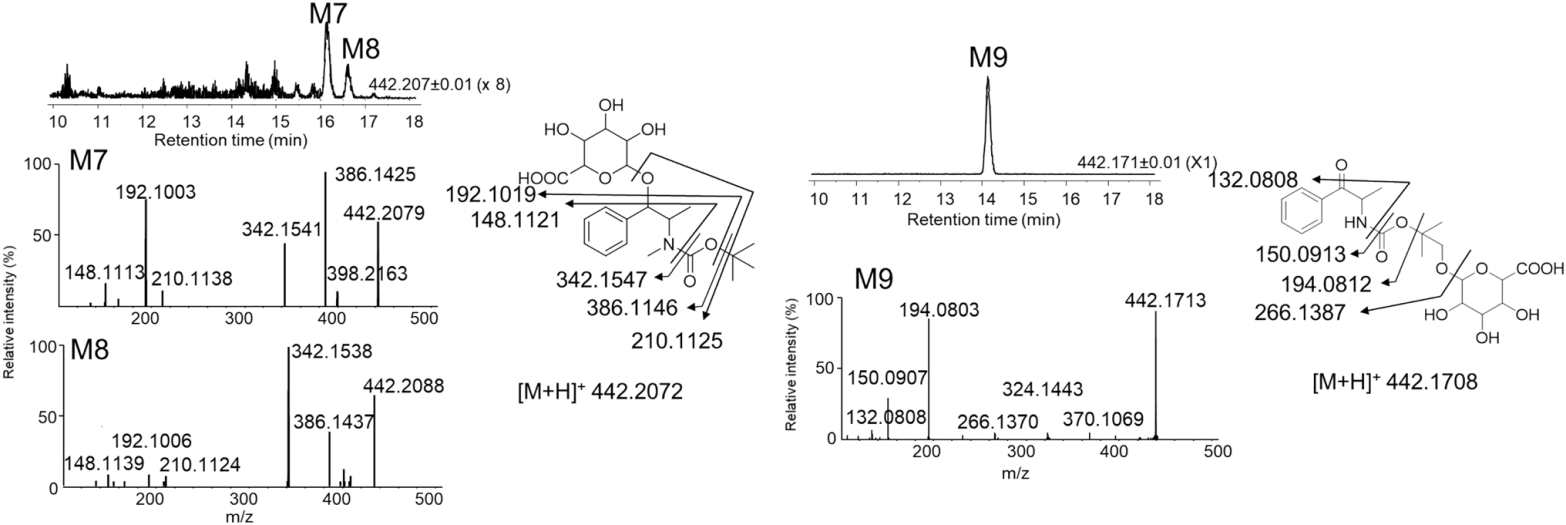
Extracted ion chromatograms and product ion spectra of M7, M8 and M9 in rat urine collected at the first 4 h form injection

To investigate the urinary excretion of BocMA and its metabolites, we conducted an administration test using rats (*n* = 3) and analyzed their urine excreted at intervals of 0–4, 4–8, 8–24, 24–48, and 48–72 h after injection using LC–QToF–MS. BocMA, M1 (D1, 2) and M5 were detected in urine from all rats, and trace amount of M3 was detected only in the urine collected during 0–4 h from Rat No. 3. M2, M4 and M6 were not detected in any of the rat urine samples. MA and its metabolites (AP, 4-OHMA, 4-OHAP) were also not detected in any of the rat urine, which corresponded with the in vitro tests. Investigation of phase II metabolites revealed three metabolite candidates, M7–9, in all of the rat urine. Their protonated molecules and product ion spectra are shown in Fig. 3, proposing M7 and M8 as glucuronides of M1 (D1, 2), and M9 as the glucuronide of M5. No sulfates of M1 and M5, or any conjugates of M3 were detected.

**Fig. 4:**
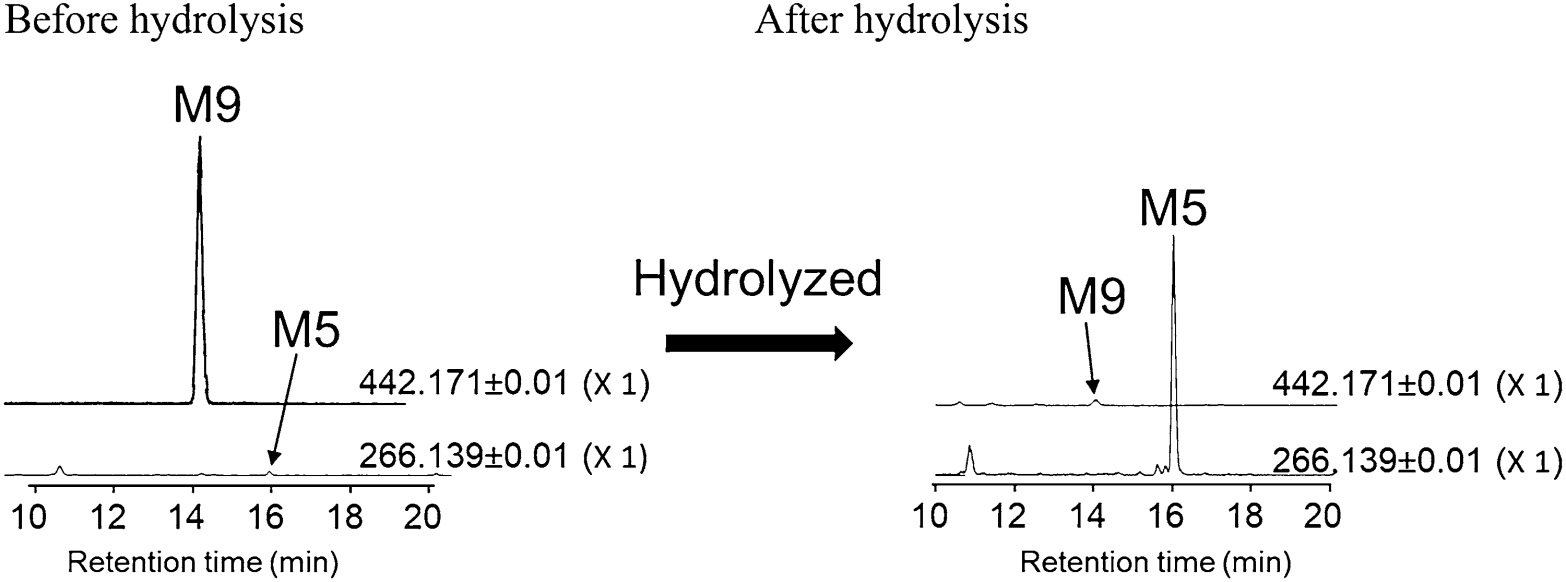
Extracted ion chromatograms of M5 and M9 obtained from rat urine before and after hydrolysis

BocMA and M1 (D1, 2) in urine were quantified for assessing the proportion of the quantity excreted in urine to the administered amount. M1 (D1, 2), partially excreted as glucuronides, was also quantified after enzymatic hydrolysis for de-conjugation. The hydrolysis condition described in the methods section was verified that it does not decompose BocMA and M1 (D1, 2) during the procedure (data not shown). Table 2 shows that BocMA concentration decreased continuously until 72 h, and the excreted BocMA as the unchanged form over 72 h accounted for 0.13% (average, *n* = 3) of the administered quantity. M1–D1 and M1–D2 concentrations before hydrolysis peaked in urine collected at 0–4 or 4–8 h (Table 2), and could not be detected in the urine collected over 24–72 h. Enzymatic hydrolysis caused M1–D1 and M1–D2 concentrations to increase and M7 and M8 to disappear. The results of M1 (D1, 2) quantification before and after hydrolysis elucidated that 90% of M1–D1 and 80% of M1–D2 (average, *n* = 3) were excreted in rat urine as glucuronides over 72 h. Urinary M1 (D1, 2) including glucuronides (M7, 8) totaled 0.016% (average, *n* = 3) of the administered amount. M5 reached the highest ion peak intensity in the urine collected over 4–8 h and decreased continuously thereafter, but was still present in the urine collected over 48–72 h. M9, the glucuronide of M5, was also detected in all rat urine collected until 72 h, reaching the highest ion peak intensity during 4–8 h. Hydrolysis also resulted in disappearance of M9 with an approximately tenfold increase of M5 peak intensity (Fig. 4). Since an authentic standard of M5 was not available, M5 concentrations were estimated on the assumption that M5 has a same detection sensitivity as M1–D1 in LC–MS/MS; urinary M5 including glucuronides (M9) over 72 h totaled 0.14% (average,* n* = 3) of the administered amount. Although M1 was proposed as a major microsomal metabolite since about half (45%) of the disappeared BocMA was metabolized into M1 (D1, 2) in the first 3 min of incubation, in vivo results revealed that only 0.016% of the administered amount was excreted in rat urine as M1 (D1, 2) including glucuronides, and also only 0.14% as M5 including glucuronide.

**Table 2:** Concentrations and total amounts of BocMA and M1 (D1, 2) excreted in rat urine within the first 72 h

Rat no.	Time (h)	Urine volume (mL)	BocMA^a^ (ng/mL)	BocMA^b^ (ng)	Before hydrolysis	After hydrolysis		Before hydrolysis	After hydrolysis		Total percentage of the excreted BocMA (%)
					M1–D1^a^ (ng/mL)	M1–D1^a^ (ng/mL)	M1–D1^b^ (ng)	M1–D2^a^ (ng/mL)	M1–D2^a^ (ng/mL)	M1–D2^b^ (ng)	
1	0–4	1.5	58	87	0.47	0.46	0.69	1.5	5.2	7.8	–
	4–8	1.0	8.9	8.9	0.51	54	54	0.67	35	35	–
	8–24	8.0	7.4	59	ND	ND	2.3	ND	ND	–	–
	24–48	7.0	12	84	ND	ND	–	ND	ND	–	–
	48–72	10	ND	–	ND	ND	–	ND	ND	–	–
	0–72	28	–	2.4 × 10^2^	–	–	57	–	–	43	0.0091
2	0–4	2.0	95	1.9 × 10^2^	6.5	5.2	10	12	12	24	–
	4–8	2.0	39	78	3.0	89	1.8 × 10^2^	1.0	14	28	–
	8–24	15	2.9	44	3.0	4.8	72	ND	ND	–	–
	24–48	20	ND	–	ND	ND	–	ND	ND	–	–
	48–72	15	ND	–	ND	ND	–	ND	ND	–	–
	0–72	54		3.1 × 10^2^	–	–	2.6 × 10^2^	–	–	52	0.015
3	0–4	0.50	5.4 × 10^3^	2.7 × 10^3^	50	1.4 × 10^2^	70	8.1	1.0 × 10^2^	50	–
	4–8	2.0	2.2 × 10^3^	4.4 × 10^3^	3.2	2.2 × 10^2^	4.4 × 10^2^	1.5	33	66	–
	8–24	10	6.8 × 10^2^	6.8 × 10^3^	ND	86	8.6 × 10^2^	ND	4.2	42	–
	24–48	8.0	83	6.6 × 10^2^	ND	11	88	ND	ND	–	–
	48–72	9.0	90	8.1 × 10^2^	ND	0.71	6.4	ND	ND	–	–
	0–72	30	–	1.5 × 10^4^	–	–	1.5 × 10^3^	–	–	1.6 × 10^2^	0.42
Average	0–72	37	–	1.6 × 10^4^	–	–	6.1 × 10^3^	–	–	85	0.15

*ND* not detected

^a^Concentration

^b^Absolute amount

## Discussion

### Metabolic pathway

**Fig. 5:**
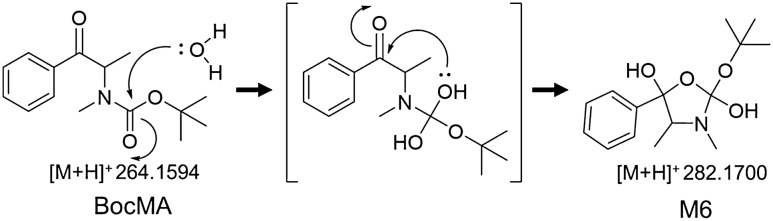
Proposed concept of BocMC hydration mechanism

**Fig. 6:**
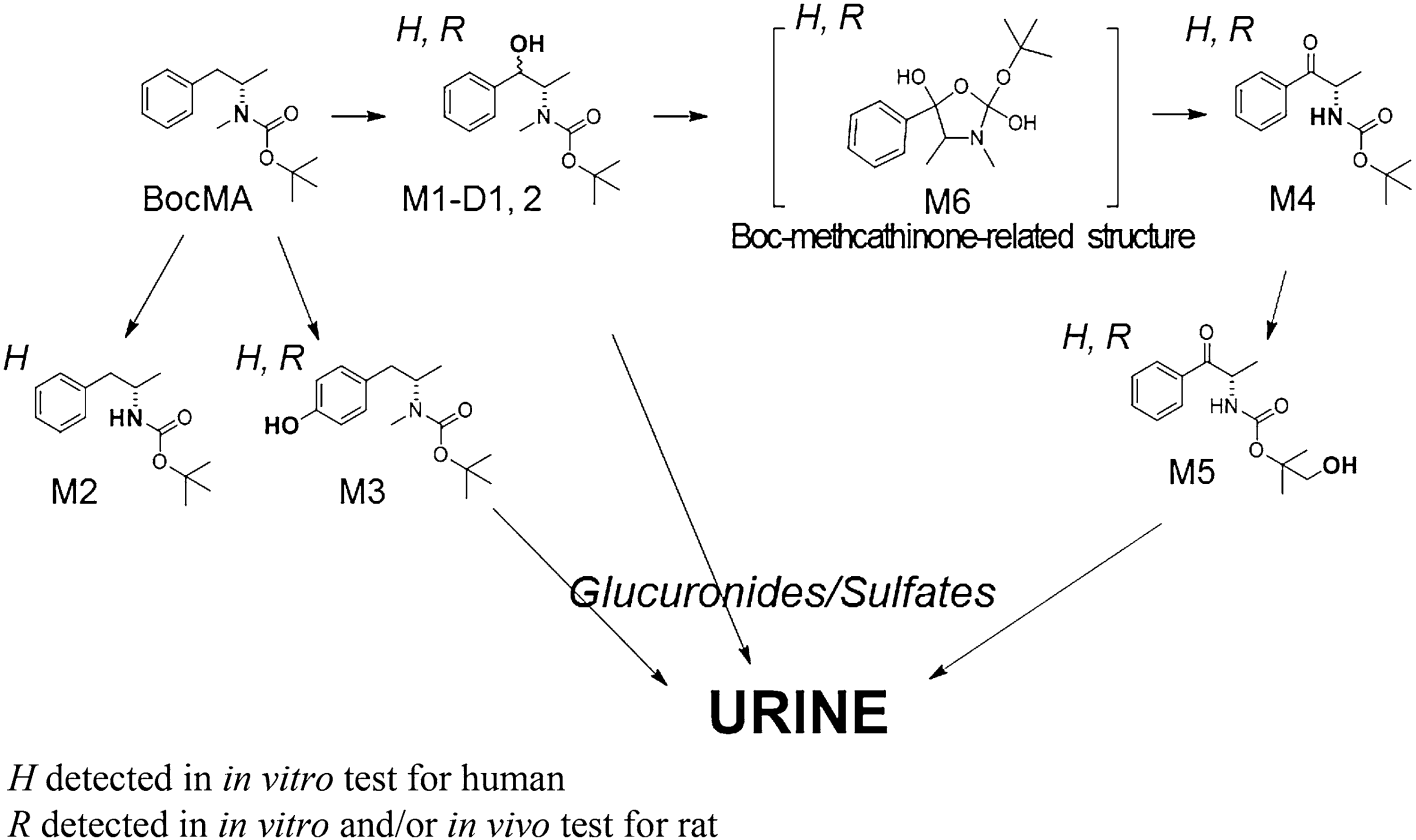
Proposed metabolic scheme of BocMA for human and rat

Microsomal study provided CL_int_, CL_H,_ and ER values indicating that BocMA experiences a significant first-pass effect after absorption following oral administration. In both human and rat metabolism, BocMA likely produces M1 (D1, 2) via hydroxylation at the benzyl position, M2 via *N-*desmethylation, M3 via hydroxylation of the phenyl group, M4 via benzylic carboxylation following *N-*desmethylation, M5 via *tert-*butylic hydroxylation of M4, and M6 as a BocMC-related compound without transformation into MA. M6 may be produced from BocMC via H_2_O adduction followed by cyclization as shown in Fig. 5 by reference to metabolism of bupropion [20–23], but this speculation still requires additional studies on structural identification and production mechanism. Chemical structures of M1, M4 and M6 proposed M1 to be further metabolized in microsomal incubation, forming M6 as an intermediate, finally yielding M4. Moreover, incubation times at which peak intensity was highest differed for each compound (BocMA and M1–6) in RLM test: 0 min for BocMA, 3 min for M1 (D1, 2), 13 min for M6, 20 min for M4, and 60 min for M5. These continuous time shifts indicated that “BocMA → M1 (D1 or 2) → M6 → M4 → M5” is one of the BocMA metabolic pathways (Fig. 6). These sequential metabolic conversions are also structurally reasonable to support the proposed pathway. Interestingly, M1 diastereomer ratio (*R* = M1–D1/M1–D2) was reversed between HLM (1 ≦ *R*≦ 3.4) and RLM (0.12 ≦ *R* ≦ 1) tests, possibly due to species difference. Such a high interspecific difference was also observed in the metabolism of *R* and *S*-bupropion which contained a *tert*-butyl group [20]. These results suggest that the steric bulkiness of the *tert*-butyl group could cause the observed species difference in stereo-selective metabolism. Additionally, reversed ratio was observed between in vitro (*R* ≦ 1) and in vivo (1 ≦ *R*) studies in rats. This result suggests that M1–D1 could be conjugated with glucuronic acid and/or excreted in urine more effectively than M1–D2.

### Urinary excretion

Urine, a non-invasively collectable biological sample, commonly contains ingested drugs and their metabolites as scientific evidence of their administration. Drug urinalysis has creditably proved illicit drug administration (i.e., MA) for police investigation; thus, it can also effectively provide valuable information for the proof of BocMA administration (and discriminate from MA administration). While our microsomal study produced seven BocMA metabolites, rat urine contained four hydroxides (M1–D1, M1–D2, M3 and M5), but not the other three metabolites (M2, M4 and M6). In rat urine, most of M1 (D1, 2) and M5 were excreted as glucuronides (M7, 8 and 9), indicating that hydroxylated metabolites were excreted more abundantly.

In addition, the total amount of BocMA and M1 accounted for only 0.15% (*n* = 3) of the administered amount, while for MA, about 80% of the administered amount is excreted in urine within 3–4 days [24]. Several highly lipophilic compounds, such as tetrahydrocannabinol and synthetic cannabinoids (the calculated lipophilicity index log P range: 2.29–8.01), hardly undergo urinary excretion likely due to tubular reabsorption [25, 26]. Our results suggest that BocMA and its in vitro major metabolites M1 which have comparable logP values (BocMA: 4.08, M1: 3.03) are also scarcely excreted in urine in such manner.

Although M3 was absent as conjugates in rat urine, it is possibly present in human urine as a sulfate because 4OHMA and 4-hydroxy-3-methoxymethamphetamine with similar phenolic hydroxyl group have been shown to be excreted in human urine predominantly as sulfates [27–30].

### Identification of BocMA administration

The present biotransformation study clarified that BocMA can produce various metabolites including diastereomers and glucuronides, but not MA and MA metabolites (AP, 4-OHMA and 4-OHAP). These results suggested the intravenously injected and inhaled BocMA do not lead to the production of MA and its metabolites in biological samples. It seems only oral intake causes BocMA to convert into MA in gastric juice [7]. Our previous report suggested the gradual degradation (half-life: 50 min in gastric juice model) will allow BocMA to undergo absorption followed by metabolism and excretion, and produce metabolites for proving its intake. From the in vitro test, microsomal metabolites (M1–6) were proposed as useful targets for the proof of BocMA intake. From the in vivo test, however, these highly lipophilic phase I metabolites were hardly or not at all excreted in rat urine but were excreted as glucuronides of the hydroxides (M7–9). Therefore, we may have to target the conjugated metabolites when analyzing an authentic human urine sample.

It is also worth noting that M1 (D1, 2) are Boc-protected pseudoephedrine, which have been reported to be seized at airports; possible intake of Boc-protected pseudoephedrine should also be considered in such cases. M3 requires additional study of urinary excretion in human because it is potentially a useful target of choice as a specific metabolite retaining the entire chemical structure of BocMA.

## Conclusion

As conversion of BocMA into MA in gastric juice can lead to misidentification of the originally ingested drug, the identification in urinalysis requires careful attention to the origin of methamphetamine. This article presented results from in vitro and in vivo studies of BocMA metabolism to find a useful target for proving BocMA intake, discovering 10 metabolites including diastereomers and glucuronides. The characterized metabolites were likely produced via phase I metabolism, such as hydroxylation, carboxylation, and demethylation, partially followed by phase II metabolism of glucuronidation. Furthermore, in vivo test revealed that lipophilic BocMA and phase I metabolites were hardly excreted in urine but instead as conjugates of the hydroxylated metabolites. This study is the first to report on the metabolism of BocMA to the best of our knowledge, and our findings provide useful information for proving BocMA ingestion alongside its discrimination from MA ingestion.
